# Effect of erythropoietin-stimulating agent on uremic inflammation

**DOI:** 10.1186/1476-9255-9-17

**Published:** 2012-05-14

**Authors:** Yuri Tanaka, Nobuhiko Joki, Hiroki Hase, Masaki Iwasaki, Masato Ikeda, Ryoichi Ando, Toshio Shinoda, Daijo Inaguma, Toshifumi Sakaguchi, Yasuhiro Komatsu, Fumihiko Koiwa, Toshihiko Yamaka, Takashi Shigematsu

**Affiliations:** 1 Division of Nephrology, Toho University Ohashi Medical Center, 2-17-6, Ohashi, Tokyo, Meguro-ku, 153-8515, Japan; 2 Department of Medicine, Division of Nephrology & Hypertension, Nephrology and Dialysis Unit, Aoto General Hospital, Jikei University School of Medicine, Tokyo, Japan; 3 Department of Nephrology, Musashino Red Cross Hospital, Tokyo, Japan; 4 Dialysis Center, Kawakita General Hospital, Tokyo, Japan; 5 Kidney Center, Nagoya Daini Red Cross Hospital, Nagoya, Japan; 6 Division of Nephrology and Blood Purification Medicine, Wakayama Medical University, Wakayama, Japan; 7 Department of Nephrology, Division of Internal Medicine, St. Luke’s International Hospital, Tokyo, Japan; 8 Division of Nephrology, Department of Internal Medicine, Showa University Fujigaoka Hospital, Yokohama, Japan; 9 Department of Clinical Engineering, Social insurance chuo general hospital, Tokyo, Japan

**Keywords:** Inflammation, CRP, Erythropoietin stimulating agent, ACE-I/ARB, Initiation of dialysis

## Abstract

**Background:**

The goal of the present study was to explore the effect of medications that are commonly prescribed for CKD patients on uremic state.

**Methods:**

This was a cross-sectional study. From January 2006 to October 2009, 1,623 patients with end-stage kidney disease (ESKD) commenced hemodialysis (HD) at the 9 participating hospitals. The criteria for exclusion from the database were 1) serum C-reactive protein (CRP) > 3 mg/dL, 2) WBC count > 9,000/mm^3^ or <4,000/mm^3^, and 3) patients with cancer, immune complex disease, or vasculitis. A total of 900 patients were entered into the final database. We explored the association of serum CRP just before the first HD session with clinical characteristics, laboratory data, and medications for CKD in the predialysis period.

**Results:**

On univariate analysis, age, CTR, eGFR, and WBC were significantly correlated with CRP. Systolic and diastolic blood pressure, serum albumin, LDL-C, HDL-C, Hb, Cr, and Ca were inversely associated with CRP. Use of erythropoietin-stimulating agents (ESA) using (r = −0.111, p = 0.0015), renin-angiotensin-aldosterone system inhibitors (r = −0.083, p = 0.0154), and calcium channel blockers (r = −0.1, p = 0.0039) was also negatively correlated with CRP. However, only use of ESA showed a significant negative correlation with CRP that was independent of other clinical factors and CKD medications on multiple regression analysis.

**Conclusion:**

ESA may strongly reduce uremic inflammation in addition to improving anemia. To confirm this potential effect, a large-scale longitudinal study would be required.

## Background

Although the mechanism of cardio-renal syndrome has been elucidated in considerable detail during the past decade [1], atherosclerotic cardiovascular disease is still the leading cause of death in patients with chronic kidney disease (CKD) [2,3]. Along with the accumulation of traditional atherogenic risk factors, factors specific to uremia, such as anemia, dyslipidemia, abnormal calcium (Ca)/phosphate (P) metabolism, insulin resistance, oxidative stress, malnutrition, and inflammation, play an important role in such rapid progression of atherosclerosis [4,5]. In particular, chronic inflammation and oxidative stress are thought to be possible treatment targets in the clinical setting [6].

According to the international guidelines [2], strict blood pressure control by using an renin-angiotensin-aldosterone (RAS) system blocker combined with other antihypertensive agents, regulation of calcium/phosphate metabolism with vitamin D or calcium therapy, and maintaining an optimum hemoglobin concentration with erythropoietin-stimulating agents (ESAs) and iron are three main essential treatments for renoprotection and a better prognosis in CKD patients. Recently, it has been suggested that medications for CKD could have possible pleiotropic effects, especially an anti-inflammatory effect. For instance, RAS blockers [7], vitamin D [8], and ESA [9] have already been shown to have anti-inflammatory activity in clinical and basic studies. However, there is still limited evidence about the effect of common treatments for CKD on inflammation in the clinical setting. The goal of the present study was to explore the effect of medications that are commonly used by CKD patients on the serum level of C-reactive protein (CRP) at the initiation of renal replacement therapy (RRT).

## Patients and methods

### Study design & patients

**Figure 1:**
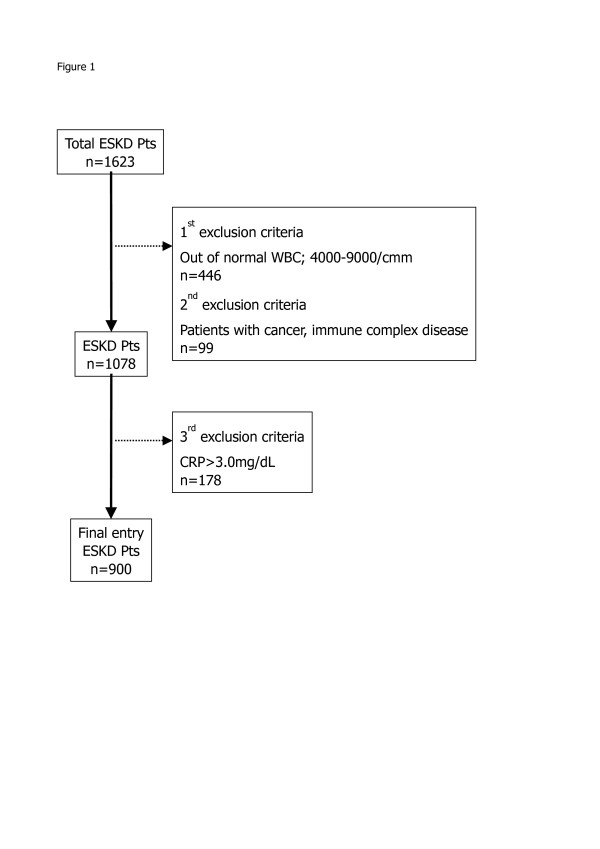
Disposition of the subjects.

We conducted a cross-sectional study using the database of the “Study Group for Assessing Initiation of Renal Replacement Therapy” (START), which includes the nephrology unit of nine institutions in Japan. The objective of START is to create a shared database on end-stage kidney disease (ESKD) patients at the time of starting RRT for the conduct of clinical research. From January 2006 to October 2009, 1,623 ESKD patients commenced chronic hemodialysis (HD) at the 9 hospitals and clinical information on those patients was added to the START database. In order to explore the anti-inflammatory effect of CKD medications, the following exclusion criteria were employed: 1) patients with an abnormal white blood cells count > 9,000/mm^3^ or <4,000/mm^3^, 2) patients who are susceptible to chronic inflammation such as those with cancer, immune complex disease, or vasculitis, and 3) in order to minimize the contamination of high CRP caused by infectious disease, the patients with a serum CRP level >3 mg/dL were also eliminated from final database according to the results of DOPPS data [10] which shows that CRP level was less than 2.5 mg/dL in 95% of Japanese stable dialysis patients. As a result, 900 ESKD patients were available for the final database of this study (Figure 1). In order to examine the factors associated with inflammation during the predialysis phase of CKD, we compared the serum CRP concentration just before the first HD session with clinical characteristics, laboratory data, and medications for CKD in the predialysis period. CKD treatments were classified as ESA, angiotensin-converting enzyme inhibitors (ACE-I), angiotensin-II receptor blockers (ARB), calcium channel blockers (CCB), other anti hypertensive agents (anti-HT), vitamin D, calcium supplements, iron supplements, and AST-120. As ESA therapy, epoetin-alfa & beta were used for renal anemia during the study period of 2006 to 2009. The last medication (AST-120) is a carbonaceous adsorbent that is used to treat CKD patients in Japan. It has been reported that AST-120 removes uremic toxins and reduces oxidative stress [11,12]. The ethics committee for clinical research of Toho University Ohashi Medical Center approved the study protocol (permission no. 13-22).

### Data collection

Clinical information was recorded on all patients at each nephrology unit immediately prior to first HD session using the same protocol. First, each patient was interviewed to obtain data on the age, gender, smoking habits, primary renal disease, previous hospitalizations, and history of hypertension, cardiac disease, cerebrovascular disease, and peripheral artery disease. We also collected information about the use of oral medications and erythropoietin during the predialysis period. Blood pressure was recorded in the supine position and a blood sample was collected just before the first HD session. The estimated glomerular filtration rate was calculated by using the new Japanese equation [13]:

(1) $$\text{eGFR}(\text{mL}/\text{min}/1.73\;{\text{m}}^{2})=194\times {\text{Cr}}^{-1.094}\times {\text{age}}^{-0.287}(\times 0.739\text{for women})\text{.}$$

The body mass index (BMI) at optimal weight was calculated as the weight in kilograms divided by the square of the height in meters. The cardiothoracic ratio (CTR) was determined at the optimal body weight after HD.

### Statistical analysis

Data are expressed as the mean ± SD. To assess the association of various factors with inflammation, Pearson’s univariate regression analysis was employed to compare clinical factors with CRP. Dummy variables were used for gender (0 for female, 1 for male), primary renal disease (0 for non-diabetic nephropathy, 1 for diabetic nephropathy), smoking history (0 for negative, 1 for positive), and each CKD treatment (0 for not used, 1 for used). The monthly ESA dose was evaluated as a numerical factor. Multivariate regression analysis was also performed to identify independent determinants of the serum CRP concentration. In all analyses, a probability (P) value < 0.05 was considered statistically significant. All statistical analyses were performed using StatView for Windows version 5.0 (SAS Institute, Cary, NC).

## Results

### Demographic characteristics of 900 new dialysis patient who enrolled into the study

The mean age was 65.5 ± 13.7 years (range: 14 to 93 years). The percentage of men (68%) and the percentage of patients with diabetic nephropathy (48%) were similar to those previously reported at the initiation of dialysis for the entire Japanese dialysis population [14]. The mean Cr and eGFR at the initiation of RRT were 9.5 ± 3.5 mg/dL and 5.4 ± 2.9 mL/min/1.73 m^2^, respectively, which were similar to the levels for the Japanese dialysis population [14]. Therefore, our database seemed to be a suitable sample of Japanese dialysis patients. The mean duration of care by a nephrologist in the predialysis phase of CKD was 33 ± 43 months. Other clinical characteristics are shown in Table 1. The mean CRP level at the initiation of RRT was used as a marker of chronic inflammation during the predialysis phase of CKD; it was 0.48 ± 0.68 mg/dL. In 133 (14.8%) of the 900 patients, the serum CRP concentration was 0.0 mg/dL, while 585 patients (65%) had a CRP in the normal range of ≤0.3 mg/dL.

**Table 1:** Clinical characteristics of the study population

**Age, years**	**65.5 ± 13.7**
Male sex (%)	611 (67.9)
BMI, kg/m^2^	23.0 ± 4.0
SBP, mmHg	153 ± 26
DBP, mmHg	78 ± 15
CTR, %	54 ± 7
Primary disease	
Chronic glomeruronephritis	226 (25.1)
Diabetic nephropathy	432 (48.0)
Glomerulosclerosis	104 (11.6)
Polycystic kidney disease	28 (3.1)
Post-renal disease	5 (0.6)
Others	105 (11.6)
Duration of nephrologist care, months	33 ± 43
WBC, /mm^3^	6160 ± 1270
Hemoglobin, g/dL	8.5 ± 1.5
Plt x 10^4^, mm^3^	19.4 ± 7.1
Albumin, g/dL	3.3 ± 0.6
LDL-C, mg/dL	98 ± 41
HDL-C, mg/dL	47 ± 17
Creatinin, mg/dL	9.5 ± 3.5
BUN, mg/dL	89 ± 27
Uric acid, mg/dL	8.6 ± 2.2
eGFR, mL/min/1.73 m^2^	5.4 ± 2.9
Calcium, mg/dL	7.8 ± 1.0
Phosphate, mg/dL	6.0 ± 1.6
Intact-PTH, pg/mL	304 ± 214
CRP, mg/dL	0.48 ± 0.68
HCO3^-^, mmol/L	18.9 ± 5.0
ESA (%)	614 (68.2)
ESA dose, u/month	17813 ± 6580
ARB (%)	489 (54.3)
ACE-I (%)	165 (16.3)
ARB or ACE-I (%)	541 (60.1)
ARB and ACE-I (%)	113 (12.6)
CCB (%)	565 (62.8)
other anti-HT drugs (%)	315 (35.0)
Vitamin D (%)	174 (19.3)
Calcium agents (%)	271 (30.1)
Iron agents (%)	85 (9.4)
AST-120 (%)	160 (17.8)

BMI; body mass index, SBP; systolic blood pressure, DBP; diastolic blood pressure, CTR; Cardiothoracic ratio, WBC; white blood cell, Plt; platelets, LDL-C; low density lipoprotein cholesterol, HDL-C; high density lipoprotein cholesterol, BUN; blood urea nitrogen, eGFR; estimated glomerular filtration rate, PTH; parathyroid hormone, CRP; C-reactive protein, ESA; erythropoietin-stimulating agents, ARB; angiotensin-II receptor blockers, ACE-I; angiotensin-converting enzyme inhibitors, CCB; calcium channel blockers, anti-HT; antihypertensive.

During the predialysis phase of CKD, about 70% of our subjects used ESA and the mean monthly dose was 17813 ± 6580 units. About 60% and 20% were using RAS inhibitor and vitamin D, respectively. Other medications are shown in Table 1.

### Univariate analysis of factors associated with CRP

Among clinical characteristics, the age (r = 0.136, p < 0.0001), CTR (r = 0.134, p = 0.0003), eGFR (r = 0.122, p = 0.0002), and WBC (r = 0.14, p = 0.0005) were significantly correlated with the serum CRP level, as shown in Table 2. Systolic blood pressure (r = −0.087, p = 0.0113), diastolic blood pressure (r = −0.068, p = 0.0484), serum albumin (r = −0.246, p < 0.0001), LDL-C (r = −0.084, p = 0.0341), HDL-C (r = −0.153, p < 0.0001), Hb (r = −0.11, p = 0.001), Cr (r = −0.102, p = 0.0023), and Ca (r = −0.118, p = 0.0004) were inversely correlated with the CRP concentration.

**Table 2:** Univariate linear regression analysis of factors correlated with the serum CRP concentration

**Variable**	**Regression coefficient**	**p value**
Age	0.136	<0.0001
Sex (F: 1, M: 2)	0.004	0.897
Diabetic nephropathy (no: 1, yes: 2)	−0.008	0.8087
BMI	0.052	0.1341
SBP	−0.087	0.0113
DBP	−0.068	0.0484
Duration of nephrologist care	−0.06	0.0775
CTR	0.134	0.0003
Albumin	−0.246	<0.0001
LDL-C	−0.084	0.0341
HDL-C	−0.153	<0.0001
Hemoglobin	−0.11	0.001
BUN	0.011	0.743
Creatinine	−0.102	0.0023
eGFR	0.122	0.0002
Uric acid	0.045	0.1861
Calcium	−0.118	0.0004
Phosphate	−0.021	0.5353
Intact-PTH	−0.007	0.8627
Platelets	0.061	0.068
WBC	0.14	<0.0001
ESA	−0.119	0.0005
ESA dose	−0.124	0.0007
ARB	−0.071	0.0361
ACE-I	−0.052	0.1282
ARB or ACE-I	−0.075	0.0261
ARB and ACE-I	−0.059	0.0841
CCB	−0.1	0.0032
other anti-HT	−0.065	0.0562
AST-120	−0.036	0.2943
Vitamin D	−0.058	0.0863
Iron agents	−0.072	0.1059

BMI; body mass index, SBP; systolic blood pressure, DBP; diastolic blood pressure, CTR; Cardiothoracic ratio, WBC; white blood cell, Plt; platelets, LDL-C; low density lipoprotein cholesterol, HDL-C; high density lipoprotein cholesterol, BUN; blood urea nitrogen, eGFR; estimated glomerular filtration rate, PTH; parathyroid hormone, CRP; C-reactive protein, ESA; erythropoietin-stimulating agents, ARB; angiotensin-II receptor blockers, ACE-I; angiotensin-converting enzyme inhibitors, CCB; calcium channel blockers, anti-HT; antihypertensive.

### Univariate analysis of CKD treatments associated with CRP

**Figure 2:**
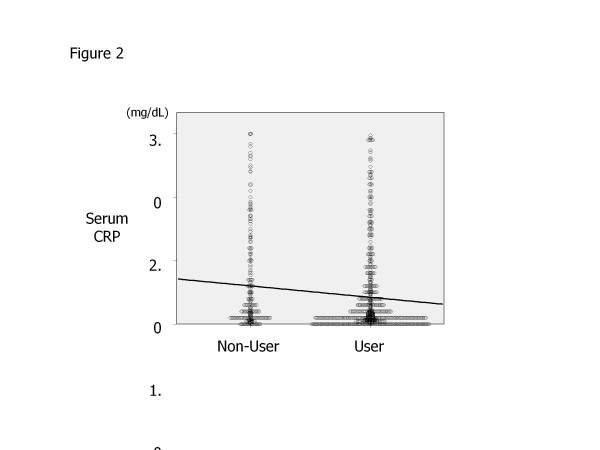
**The CRP levels of ESKD patients with and without ESA using at the initiation of renal replacement therapy.** Significantly less CRP concentration was found in ESA user compared with non-user by using student’s *t*-test (0.42 ± 0.65 mg/dL vs. 0.61 ± 0.76 mg/dL). Solid line depicts the correlation coefficient by means of Pearson’s univariate regression analysis (r = −0.119, p = 0.0005).

Because the duration of care by a nephrologist before starting RRT was thought to influence the association of CKD treatments with CRP, all treatments were adjusted by the “duration of nephrologist care,” which did not show a significant correlation with CRP (r = −0.06, p = 0.0775). As shown in Table 3, use of ESA (r = −0.111, p = 0.0015) and the dose of ESA (r = −0.117, p = 0.0017), use of ARB (r = −0.076, p = 0.0275), use of ARB or ACE-I (r = −0.083, p = 0.0154) (corresponding to use of RAS-I), and use of CCB (r = −0.1, p = 0.0039) were negatively correlated with the CRP level. Strongest inverse association was observed between CRP and ESA using, as shown in Figure 2.

**Table 3:** **Linear regression analysis of the association between CKD treatments and the serum CRP concentration**^**#**^

**Factor**	**Regression coefficient**	**95%CI**	**p value**
ESA	−0.111	−0.274 - -0.065	0.0015
ESA dose	−0.117	−0.014 - -0.003	0.0017
ARB	−0.076	−0.196 - -0.012	0.0275
ACE-I	−0.061	−0.221 - 0.012	0.0785
ARB or ACE-I	−0.083	−0.212 - -0.022	0.0154
ARB and ACE-I	−0.065	−0.267 - 0.005	0.0589
CCB	−0.1	−0.241 - -0.046	0.0039
other anti-HT	−0.068	−0.188 - 0.001	0.0515
AT-120	−0.041	−0.190 - 0.046	0.2306
Vitamin D	−0.064	−0.220 - 0.007	0.0664
Fe	−0.086	−0.334 - 0.003	0.054

# All factors are adjusted by the "duration of nephrologist care"

ESA; erythropoietin-stimulating agents, ARB; angiotensin-II receptor blockers, ACE-I; angiotensin-converting enzyme inhibitors, CCB; calcium channel blockers, anti-HT; antihypertensive.

### Multivariate analysis of CKD treatments associated with CRP

In order to examine whether any CKD treatments were independent determinants of CRP, we performed further analyses using multivariate linear regression models (Table 4). In the first model, ESA and CCB showed an independent inverse correlation with CRP among CKD treatments, while use of RAS-I was not selected. In the second model, age and eGFR were added to the first model, after which use of ESA and CCB still showed a significant association with CRP. For the third model, the additional covariates of albumin, LDL-C, HDL-C, and Hb were added to the first model. Use of RAS-I showed an independent association with CRP instead of CCB, while ESA maintained a strong inverse correlation with CRP. For the fourth model, sBP and CTR were added to the first model, and ESA and CCB were selected again. Finally, all of the factors were included in the fifth model and significant correlations of ESA and RAS-I with CRP were observed. These findings suggested that only use of ESA had a significant inverse correlation with the serum CRP concentration.

**Table 4:** Multiple linear regression analysis of factors associated with the serum CRP concentration

	**Model 1**			**Model 2**			**Model 3**			**Model 4**			**Model 5**		
**Variable**	**Regression coefficient**		**p value**	**Regression coefficient**		**p value**	**Regression coefficient**		**p value**	**Regression coefficient**		**p value**	**Regression coefficient**		**p value**
	**Lower**	**Upper**		**Lower**	**Upper**		**Lower**	**Upper**		**Lower**	**Upper**		**Lower**	**Upper**	
Age				0.124		0.0003							0.112		0.0115
				0.003	0.01								0.001	0.01	
SBP										-0.087		0.0228	-0.099		0.0249
										-0.004	-0.0003				
CTR										0.134		0.0005	0.011		0.8073
										0.006	0.021		-0.008	0.01	
Albumin							−0.262		<0.0001				-0.27		<0.0001
							−0.423	−0.224					-0.43	-0.219	
LDL-C							−0.122		0.0027				-0.107		0.013
							−0.003	−0.001					-0.003	0.0004	
HDL-C						−0.148			0.0003				-0.141		0.0009
						−0.009		−0.003					-0.009	-0.002	
Hemoglobin							−0.043		0.2889				-0.079		0.0761
							−0.059	0.018					-0.078	0.004	
eGFR				0.09		0.0108							0.077		
				0.005	0.036								-0.002	0.038	0.084
ESA	−0.094		0.0086	−0.075		0.0348	−0.131		0.0014	-0.078		0.0447	-0.116		0.0072
	−0.253	−0.037		−0.225	−0.008		−0.354	−0.085		-0.244	-0.003		-0.335	-0.053	
ARB or ACE-I	−0.054		0.1229	−0.055		0.1159	−0.098		0.0157	-0.054		0.1589	-0.095		0.0258
	−0.175	0.021		−0.175	0.019		−0.266	−0.028		-0.184	0.03				
CCB	−0.088		0.0129	−0.09		0.0097	−0.08		0.052	-0.097		0.0133	-0.077		0.0732
	−0.228	−0.027		−0.23	−0.032		−0.247	0.001		-0.249	-0.029		-0.246	0.011	

SBP; systolic blood pressure, CTR; Cardiothoracic ratio, LDL-C; low density lipoprotein cholesterol, HDL-C; high density lipoprotein cholesterol, eGFR; estimated glomerular filtration rate, ESA; erythropoietin-stimulating agents, ARB; angiotensin-II receptor blockers, ACE-I; angiotensin-converting enzyme inhibitors, CCB; calcium channel blockers.

## Discussion

It is well established that chronic inflammation plays a crucial role in the progression of uremic atherosclerotic cardiovascular disease in CKD patients [15-17]. There is increasing evidence of a relationship between the serum level of CRP, a marker of chronic inflammation, and cardiovascular disease in these patients [15,18]. The present study demonstrated that at the initiation of RRT 1) 35% of ESKD patients had abnormally high CRP levels; 2) CRP was positively correlated with age, CTR, and eGFR, and negatively correlated with blood pressure, albumin, HDL-C, HDL-C, and Hb; 3) use of ESA showed an independent inverse correlation with CRP. These findings imply that use of ESA in the predialysis phase of CKD has an anti-inflammatory effect and would be beneficial for prevention of progression of atheroscletic complications.

Chronic inflammation is thought to be one of the central reasons for the high incidence and prevalence of atherosclerotic cardiovascular disease in CKD patients [16]. CRP is a well known marker of inflammation in the general population [19]. In CKD patients, it has been reported that CRP is closely associated with the severity of atherosclerosis and cardiovascular events [17,18]. Indeed, several reports have suggested that CRP could be associated with atherosclerosis through various mechanism such as 1) release of reactive oxygen species, 2) increased expression of adhesion molecules, 3) induction of foam cell formation, and 4) destabilization of plaque [6]. If CRP is involved in the pathophysiology of cardiovascular disease, it could be expected that reducing the CRP level would prevent the development of cardiovascular complications. In the present study, use of ESA, RAS-I, and CCB during the predialysis period showed a significant inverse correlation with serum CRP. Although we do not have any information about the duration of such treatments in the predialysis phase of CKD, the effect of each medication on CRP remained significant after adjustment for the duration of care by a nephrologist. This may imply that ESA, RAS-I, and CCB have an anti-inflammatory effect in the predialysis phase of CKD which is independent of the duration of treatment.

Treatment with RAS-I is well known to reduce chronic inflammation in CKD patients. In the current study, 60% of the subjects were on treatment with RAS-I in the predialysis period and most of them received ARB. At least 6 ARB are recognized to have an anti-inflammatory effect and have been shown to reduce CRP in clinical studies. Dohi et al [20]. reported that candesartan treatment for 12 weeks decreased the serum level of CRP by 14% in patients with essential hypertension. In addition, valsartan therapy for 8 months reduced CRP by 39% in hypertensive patients with left ventricular hypertrophy [21] or with other cardiovascular risk factors [22], while 3 months of irbesartan therapy for patients with coronary artery disease significantly reduced the plasma CRP level [23]. A large-scale prospective, double-blind, placebo-controlled, multicenter study performed in patients with essential hypertension and microinflammation also showed that olmesartan therapy significantly reduced the serum level of CRP [24]. Moreover, reduction of serum CRP was observed after losartan was administered for 28 days to patients who had interstitial inflammation and fibrosis associated with chronic cyclosporine-induced nephropathy [25]. Finally, treatment with telmisartan for 3 months reduced the serum level of CRP by 38% in diabetic patients [26]. It is interesting that irrespective of the underlying disease and the duration of treatment, an anti-inflammatory effect of ARB therapy was confirmed. These reports support our finding that the beneficial anti-inflammatory effect of ARB was still detectable after adjusting for the duration of care by a nephrologist. There have also been several studies into the effects of ACE-I on CRP in various diseases [26-28].

Compared with ARB, less is known about the clinical effect of CCB on CRP, but a few studies have clearly shown a suppressive effect of CCB on inflammation. After 3 months of CCB treatment, reduction of the serum CRP level was observed in patients with vasospastic angina [29]. Also, the combination of a RAS-I with the CCB azelnidipine decreased serum CRP more than combined therapy with other CCB [30]. This implies that the effect on CRP may depend on the type of CCB. Unfortunately, no information concerning the type of CCB was included in our database, so we could not investigate this issue.

Intriguingly, use of ESA was strongly associated with a lower CRP level in the current study. This association remained even after adjusting for RAS-I and CCB, as well as other variables. Several other reports support our finding of an inverse association between use of ESA and the serum level of CRP. To our knowledge, Agnello et al. first identified a possible anti-inflammatory effect of ESA in an experimental rat model of autoimmune encephalomyelitis [9]. They showed that ESA decreased the peak clinical severity of experimental autoimmune encephalomyelitis in a dose-dependent manner, with a simultaneous marked decrease of TNF and the pro-inflammatory cytokine IL-6 in the spinal cord. In the clinical field, Kourea et al [31]. demonstrated in their single-blind, placebo-controlled trial that patients with anemic congestive heart failure who received combined treatment using the erythropoietin analogue darbepoietin-α and oral iron had a greater increase of Hb than those treated with placebo plus oral iron, and also showed concomitant improvement of cardiac contractility. As a possible mechanism of this cardioprotective effect, they confirmed an approximately 40% decrease of IL-6 (a pro-inflammatory cytokine) by treatment with darbepoietin-α, compared with an increase of about 20% after placebo treatment. This finding suggests that ESA has an anti-inflammatory effect in patients with sustained inflammation such as those with heart failure. Since it is well known that IL-6 regulates the hepatic synthesis of CRP, our main finding that CRP was lower in patients using ESA compared to those without ESA agrees with the above evidence.

The most popular class of medication with anti-inflammatory and pleiotropic effects is the statins. Even in patients with end-stage kidney disease, CRP is decreased by 11.5% as a result of statin therapy [32]. However no clinical advantage of statin treatment has been identified in ESKD patients [32,33]. Because there is little evidence of a beneficial effect of statin therapy for advanced CKD, statins are still not standard treatment for CKD patients, especially those in stages 4 and 5. Therefore, we did not include information on the use of statins in our database.

Because the current study had a cross-sectional design, this is a limitation, since we did not have any information about the duration of each treatment in the predialysis phase, apart from the duration of care by a nephrologist. Based on the hypothesis that there would be a close association between nephrologist care and treatment for the predialysis phase of CKD, all treatments were first adjusted by the duration of care. We consider that this minimized the potential bias. The second limitation of our study is that the marker of chronic inflammation in the predialysis phase of CKD was the serum CRP level measured just before the first dialysis session. We could not completely exclude the possibility that this CRP level did not reflect chronic inflammation in our uremic patients, although we excluded patients with suspected infection (defined as those with an abnormal WBC count). We also excluded patients who are susceptible to latent chronic inflammation, including those with cancer, immune complex disease, or vasculitis, from our final database. However, such exclusions could have narrowed the focus of our investigation.

In conclusion, the present cross-sectional study explored the potential anti-inflammatory effect of common CKD medications in the real world clinical setting. We found that use of ESA had the strongest connection with lower CRP levels. Accordingly, ESA may reduce uremic-specific inflammation in addition to the main effect of these agents on anemia. To confirm these findings, a large-scale longitudinal study will be required.

## Competing interests

The authors declare that they have no competing interests.

## Authors’ contributions

This manuscript has not been published and is not under consideration for publication elsewhere. All the authors have read the manuscript and have approved this submission.
